# Use of the term whole grain on the label of processed and ultra-processed foods based on cereals and pseudocereals in Brazil

**DOI:** 10.3389/fnut.2022.875913

**Published:** 2022-08-15

**Authors:** Érika Arcaro Bez Batti, Amanda Bagolin do Nascimento, Ana Paula Gines Geraldo, Ana Carolina Fernandes, Greyce Luci Bernardo, Rossana Pacheco da Costa Proença, Paula Lazzarin Uggioni

**Affiliations:** ^1^Nutrition Postgraduate Program, Health Sciences Centre, Federal University of Santa Catarina (UFSC), Florianópolis, Brazil; ^2^Nutrition in Foodservice Research Centre (NUPPRE), Health Sciences Centre, Federal University of Santa Catarina (UFSC), Florianópolis, Brazil

**Keywords:** whole grain, whole foods, ingredients list, food labeling, packaged foods

## Abstract

There has been an increasing consumption of processed and ultra-processed foods, accompanied by growing concerns about the relationship between diet quality and health. Whole-grain foods, composed of cereals and pseudocereals, are recommended as part of a healthy diet, and food labeling is an important tool for consumers to identify the presence of whole grains in packaged foods. This study aimed to analyze the use of the term whole grain on the label of processed and ultra-processed foods based on cereals and pseudocereals (amaranth, quinoa, and buckwheat) in Brazil. Data were collected by a census of all food labels in a Brazilian supermarket. Foods were classified into eight groups according to Brazilian legislation and according to the presence or absence of the term whole grain. The prevalence of foods displaying the term whole grain or related expressions on the front label was assessed, and differences between groups were analyzed using Pearson's chi-squared test. Comparisons were also made in relation to the position of whole-grain ingredients in the ingredients list, given that Brazilian food labeling regulations require that ingredients be listed in descending order of weight on packaged foods. The level of significance was defined as *p* < 0.05. The sample included 1,004 processed and ultra-processed foods based on cereals and pseudocereals, 156 (15.6%) of which displayed the term whole grain and/or similar expressions on the front label. Of these, 98 (9.8%) contained the term whole grain, 25 (2.5%) displayed analogous expressions, and 33 (3.3%) contained the term whole grain concomitantly with analogous terms, identified in foods of the groups Bakery goods, bread, cereals, and related products and Sugars, sugary foods, and snacks. Half of the food products displaying the term whole grain or related expressions on the front label did not have a whole-grain ingredient listed in the first position of the ingredients list. The frequency of whole grains was even lower when analyzing the second and third ingredients. These findings reveal the existence of inaccurate information regarding the term whole grain or analogous expressions on the front label of cereal- and pseudocereal-based packaged foods. It is expected that these results will contribute to stimulating the food industry and regulatory bodies to improve the use of the term whole grain and related expressions on packaged food labels, given that, up to the moment of data collection, there were no regulatory requirements for these statements. Furthermore, the findings might contribute to improving the clarity of information available on food labels, thereby preventing consumer deception at the time of purchase.

## Introduction

The NOVA classification system groups foods into four categories according to the degree and type of processing to which they are subjected: (i) fresh and minimally processed foods, (ii) processed culinary ingredients, (iii) processed foods, and (iv) ultra-processed foods (1). The replacement of fresh and minimally processed foods for ultra-processed foods with high energy density and low nutritional quality has been associated with a global increase in obesity and other chronic non-communicable diseases (2, 3).

The 2017–2018 Consumer Expenditure Survey (POF), conducted by the Brazilian Institute of Geography and Statistics (IBGE) and based on the NOVA classification, revealed that the purchase of fresh and minimally processed foods is decreasing, whereas the purchase of processed and, mainly, ultra-processed foods is increasing among the Brazilian population (4). Processed foods are defined as industrial products prepared by adding salt, sugar, oils, or fats and by using preservation methods, such as canning and bottling, to increase durability. Most processed foods have two or three ingredients. Ultra-processed foods are ready-to-eat industrial formulations that typically contain five or more ingredients and additives that intensify visual or sensory aspects, such as dyes, emulsifiers, and flavorings (1, 5).

The relationship between diet quality and health has been widely discussed in recent years. Whole foods have attracted great attention in this context because they are good sources of nutrients such as dietary fibers, vitamins, minerals, and phytochemicals (6–9). Of note, there is the notion that whole-grain foods have higher fiber and nutrient contents. However, this relationship should be analyzed with care. Analysis of fiber content is not a reliable method to assess whether a food product is whole grain, given that fibers from other sources may be added (10). For example, foods containing refined wheat flour and added fibers will not have the same levels of vitamins, minerals, or phytochemicals as similar foods prepared with whole grains because refinement may cause significant losses in nutrients (11–16).

Given that the consumption of whole grains is recommended as part of a healthy diet (8, 11, 17–19), government bodies and health promotion organizations worldwide have included this recommendation in their dietary guidelines (11, 18, 20). Definitions of whole grain are varied. Countries such as Australia, New Zealand, Singapore, Canada, Mexico, Denmark, Norway, and Sweden define whole grains as grains containing all major components in the same proportions as those found in the entire seed; however, countries differ in their requirements regarding the types of cereals or pseudocereals that qualify as grains and the processing methods that can be used to obtain whole grains (12, 13, 21–27). Likewise, production and labeling requirements for whole-grain foods differ greatly between countries. For instance, bread must contain 90% whole grains to be labeled as whole grain in Germany. In Canada, whole-grain bread must contain at least 60% whole wheat flour in relation to the total amount of flour used. In Scandinavian countries (Denmark, Norway, and Sweden), the requirement is a minimum of 50% whole grains on a dry weight basis (28–30).

In Brazil, there is no legal definition or quantitative or qualitative criteria to identify whole-grain products. Despite this gap, the Brazilian Health Regulatory Agency (ANVISA) expressed concern that many manufacturers misuse the term whole grain on food labels, perhaps in an attempt to convey the impression that the product is healthier (31). This inaccurate information can deceive consumers as to the true composition of food products, influencing food choices in a misleading way (10).

Regardless of the definition of whole grain, food labeling represents the primary means of informing consumers about the composition of packaged foods. Information displayed on food labels can guide consumers in selecting a food product, assessing its content, and understanding its proper use (32–35).

This study aimed to analyze the use of the term whole grain on the label of processed and ultra-processed foods based on cereals and pseudocereals in Brazil. To date, we identified five studies that analyzed claims, labels, and ingredients lists of food products formulated with whole grains, with special emphasis on whole-grain breads, cakes, cereal bars, and biscuits (36–40). Three of these studies were conducted in Brazil and analyzed consumer perceptions, nutrition information, and the ingredients list of whole breads and biscuits according to current regulatory requirements (37, 38, 40). However, no study was found to analyze all whole-grain foods available for sale at a given time and place. The present research collected data from a supermarket located in southern Brazil. Although limited, this method provided an overview of food items sold in a conventional supermarket and of products broadly available in the country.

## Materials and methods

### Study design

This cross-sectional study investigated all retail packaged food products available in a large Brazilian supermarket. The supermarket was chosen purposely and belongs to one of the 10 largest Brazilian supermarket chains, which has 27 stores throughout the country, according to the Brazilian Supermarket Association (41). Most of the food and beverage products sold in this supermarket are of broadly available brands and can be found in other large supermarkets throughout the country.

A survey was performed by mapping all aisles in the store. Data collectors were responsible for mapping every product in assigned aisles. All ready-for-sale products, regardless of their origin, that were packed in the absence of the consumer are subject to the Brazilian and Mercosur Regulation on Food Labeling (No. 360/2003) and were included in the audit (42). The supermarket manager gave written consent for the research.

### Data collection

Data were gathered over a 3-month period (October to December 2013) in Florianópolis, Santa Catarina State, southern Brazil. Information on product name and type, nutritional values (energy, carbohydrate, protein, total fat, saturated fat, trans fat, fiber, and sodium contents per serving), and serving sizes were obtained in-store from product labels. These data were then fed into an electronic version of the data collection form developed by Kliemann et al. (43). The electronic form was created using Epi Collect Plus software and then installed on tablet computers. All food labels were photographed in-store to record their ingredients lists, which were later transcribed to the electronic form. This procedure was adopted so as not to disturb shoppers, given that transcribing requires a long time to be executed. For data collection, we recruited graduate and undergraduate Nutrition students (*n* = 12), who received practical-theoretical training and, 1 month before data collection, participated in a field test in a different supermarket.

To improve quality control of data, we evaluated, as in a previous study (44), the inter-rater agreement between photographs and data transcribed to the electronic forms for 5% of the products. Kappa test results showed a high degree of inter-rater agreement between the two databases (>99%, *p* < 0.05).

### Data management

Collected data were transferred directly from tablet computers to an online database at the end of each collection day. The database was exported to a Microsoft Excel 2010 spreadsheet. Each food product was coded with a number, and, later, each image in the database was renamed to the corresponding food product number.

### Identification and selection of cereal and pseudocereal foods labeled or not as whole grain

Brazilian legislation (45) classifies and numbers labeled foods into eight groups, as follows: (I) Baking goods, breads, cereals, legumes, roots, tubers, and related products; (II) Fresh and canned vegetables; (III) Fruits, juices, syrups, and drink mixes; (IV) Milk and dairy products; (V) Meat and eggs; (VI) Oils, fats, and nuts; (VII) Sugars and products in which carbohydrates and fats are the main energy sources; and (VIII) Gravies, sauces, ready-made seasonings, broths, soups, and ready-to-eat dishes.

We analyzed which groups contained foods based on cereals and pseudocereals. For the purposes of this study, cereals included canary grass, rice, wild rice, oat, rye, barley, fonio, adlay, common millet, pearl millet, corn, sorghum, teff, wheat, and triticale and pseudocereals included amaranth, quinoa, and buckwheat (6, 46–50).

We chose to assess processed and ultra-processed foods because they are generally formulated with various ingredients, and grains may not be used in their intact form. Groups containing only fresh and/or minimally processed foods and/or foods not based on cereals or pseudocereals were excluded from the analysis.

After defining food groups, we identified processed and ultra-processed foods based on cereals and pseudocereals, according to food census data (*n* = 5,620) (44). A decision flowchart was used to identify the degree of processing (51). Then, photographs of the front-of-pack of food items were analyzed for identification of whole grain or similar terms. Analogous terms were defined as any expression containing the term “whole” as part of the label statement (e.g., “with whole flour” and “made with whole grain”) but not as part of a claim or product name.

We developed a flowchart to assist in food identification (Supplementary material). This tool contains dichotomized questions to determine whether a food item should be included or excluded from the analysis. Food products were classified according to the presence of whole-grain-related terms: category 1, products containing the term whole grain; category 2, products containing analogous terms; category 3, products containing both whole grain and analogous terms; and category 4, conventional products that did not contain whole grain or analogous terms.

Similar foods were defined as those that were of the same group as whole-grain foods but lacked these terms, being possible to infer from the front label that these products contained refined cereals (for example, a sliced bread product containing the term traditional on the front-of-pack label). It is noteworthy that the process of regulating food products with traditional claims in the global market is still incipient. No regulatory mechanisms were found for the use of traditional claims. The United Kingdom Food Standards Agency published guidelines concerning commonly misused terms in food labels, including the descriptor traditional. The main objectives of the document were to help food manufacturers and producers decide when to use these marketing terms, help enforcement authorities identify inappropriate uses, and help consumers make informed choices (52). In Brazil, legislation states that food labels should not contain misleading information on the nature, composition, origin, or quality of the food product (53). Thus, it can be understood that packaged foods should not be described as traditional or contain related terms.

### Assessment of the ingredients list

We identified ingredients derived from cereals and pseudocereals (e.g., whole cereal/pseudocereal or broken, cracked, and flaked derivatives) through qualitative analysis of food labels. Cereal and pseudocereal ingredients were classified as simple or compound (e.g., flour and mixture of flours, respectively). Subsequently, the ingredients were further classified as refined or whole grain. Whole grain items were identified as whole cereals, whole pseudocereals, or items containing the word “whole” in their name. It should be noted that compound ingredients were considered whole grain only when all ingredients were whole grain (e.g., mixture of whole flours).

### Data analysis

Assessment of the use of whole-grain-related terms on the labels of processed and ultra-processed foods containing cereals and pseudocereals was carried out using two indicators: prevalence of whole grain and similar terms on the front-of-pack and frequency of whole cereals among the first three ingredients in the ingredients list of processed and ultra-processed foods containing whole-grain-related terms. For this purpose, descriptive statistical analyses were performed, and results are expressed as prevalence.

Pearson's chi-squared test was used to assess differences in the prevalence of the use of whole grain or similar terms between food groups (45).

We carried out a descriptive analysis of the ingredients list of processed and ultra-processed foods to identify whether whole grains were the main ingredients. The order of cereal- and pseudocereal-based ingredients in ingredients lists was analyzed using Microsoft Excel 2019 spreadsheets. It should be clarified that, according to Brazilian and Mercosur legislation, ingredients must be listed in descending order of proportion by weight (53). Therefore, the relative amount of whole-grain ingredients in a food product can be deduced from their position in the ingredients list. Whole grains should be the first or second ingredient, preferably listed after water only; for foods containing several whole-grain ingredients, these ingredients should appear at the beginning of the list (54, 55). Therefore, we intentionally analyzed the presence of whole grains among the first three items of the ingredients list. Descriptive statistical analyses were performed, and results are expressed as prevalence.

Differences in the prevalence of whole grain or similar terms between food groups were assessed at the 5% significance level. Data analysis was performed using Stata version 13.0 (56).

## Results

We analyzed the labels of 1,004 processed and ultra-processed foods based on cereals and pseudocereals. According to Brazilian legislation (45), these foods were categorized into two groups, namely group I, Bakery goods, bread, cereals, and related products (*n* = 578), and group VII, Sugars, sugary foods, and snacks (*n* = 426). Group VIII (Gravies, sauces, ready-made seasonings, broths, soups, and ready-to-eat dishes) was excluded from the analysis because whole-grain-related terms were only identified in fresh or minimally processed foods (e.g., almost ready-to-eat mixture of cereals and legumes). The other groups described by legislation were not included in the analysis because they did not contain foods based on cereals or pseudocereals (groups II, III, IV, V, and VI).

A total of 156 (15.5%) food products exhibited whole-grain-related terms, of which 98 (9.8%) displayed the term whole grain, 25 (2.5%) exhibited analogous terms, and 33 (3.3%) had whole grain and analogous terms on the front-of-pack (Table 1). In group I, 87 (15.1%) foods exhibited the term whole grain, 25 (4.3%) exhibited analogous terms, and 22 (3.8%) exhibited both whole grain and analogous terms. In group VII, 11 (2.6%) food products displayed the term whole grain and 11 (2.6%) displayed both whole grain and analogous terms.

**Table 1:** Description and prevalence of processed and ultra-processed foods based on cereals and pseudocereals labeled or not as whole grain or related terms, stratified into food groups according to Brazilian Resolution No. 359/2003.

**Category 1: Foods**		**Category 2: Foods**		**Category 3: Foods**		**Category 4: Similar**		**Total *N* (%)**
**labeled as**		**labeled with**		**labeled as whole**		**conventional**		
**whole grain**		**related terms**		**grain plus related terms**		**foods**		
***n* (%)**	**Examples**	***n* (%)**	**Examples**	***n* (%)**	**Examples**	***n* (%)**	**Examples**	
Group I - Bakery goods, bread, cereals, and related products								
87 (15.1)	Whole-grain biscuit, whole-grain noodles, whole-grain rye bread, whole-grain toast	25 (4.3)	Breakfast cereal with whole grains, granola with five whole grains, bread mix with whole flour	22 (3.8)	Salted whole-grain biscuits with rye and whole grains, whole-grain bread made with 100% whole-grain flour	444 (76.8)	Salted biscuits, cakes without filling, breakfast cereal, bread	578 (100)
Group VII - Sugars, sugary foods, and snacks								
11 (2.6)	Whole-grain cake, whole grain panettone, whole-grain cookies	–	–	11 (2.6)	Whole milk and honey biscuits made with whole grains, whole-grain cake with whole flour	404 (94,8)	Sweet cookies, cakes with filling, panettone	426 (100)
**98 (9.8)**	**–**	**25 (2.5)**	**–**	**33 (3.3)**	**–**	**848 (84.4)**	–	**1,004 (100)**

Regarding group I, the food products that were most frequent in category 1 were salted biscuits (48.3%); in category 2, granola (48.0%); and, in category 3, salted biscuits and breads (40.9% each). The most frequent foods of group VII in category 1 were corn snacks (45.5%) and, in category 3, cookies (45.5%) (Table 2).

**Table 2:** Examples of processed and ultra-processed foods based on cereals or pseudocereals exhibiting whole grain and/or analogous terms on the front label, stratified into food groups according to Brazilian Resolution No. 359/2003.

**Type of food**	**Category 1: Foods labeled as whole grain**	**Category 2: Foods labeled with related terms**	**Category 3: Foods labeled as whole grain plus related terms**
	***n* (%)**	***n* (%)**	***n* (%)**
Group I - Bakery goods, bread, cereals, and related products			
Salted biscuits	42 (48.3)	–	9 (40.9)
Bread	27 (31.0)	–	9 (40.9)
Granola	7 (8.0)	12 (48.0)	–
Empanada	3 (3.4)	–	–
Pasta	2 (2.3)	–	–
Cereal bars	2 (2.3)	2 (8.0)	–
Bread mix	1 (1.1)	1 (4.0)	–
Cake mix	1 (1.1)	–	–
Breakfast cereal	1 (1.1)	10 (40,10)	2 (9.1)
Waffle	1 (1.1)	–	–
Cake without filling	–	–	2 (9.1)
Group VII - Sugars, sugary foods, and snacks			
Corn snacks	5 (45.5)	–	1 (9.1)
Cookies	4 (36.4)	–	5 (45.5)
Panettone	1 (9.1)	–	–
Sweet popcorn	1 (9.1)	–	–
Sweet biscuits	–	–	4 (36.4)
Cake with filling	–	–	1 (9.1)

In group I, of the 87 food products containing the term whole grain (category 1), in 25 (28.7%), the term was used as a claim, in 12 (13.8%), the term was used as a product name, and, in 50 (57.5%), the term was used both as a claim and as a product name. In foods of category 3, whole grain was used as a product name in 15 products (68.2%) and as a claim and product name in seven foods (31.8%). Of category 3 foods that used whole grain as a claim and product name, only six products (27.3%), including breads, exhibited the percentage of whole-grain cereals on the front label (Table 3).

**Table 3:** Use of whole grain expressions on the front label of processed and ultra-processed foods based on cereals or pseudocereals, stratified into food groups according to Brazilian Resolution No. 359/2003.

**Food group**	**Category**	**Whole-grain term used as claim**	**Whole-grain term used as product name**	**Whole-grain term used as claim and product name**	**Total**
		***n* (%)**	***n* (%)**	***n* (%)**	***N* (%)**
	Category 1: Foods labeled as whole grain	25 (28.7%)	12 (13.8)	50 (57.5)	87 (100)
I^a^	Category 3: Foods labeled as whole grain plus related Terms	–	15 (68.2)	7 (31.8)	22 (100)
	Category 1: Foods labeled as whole grain	9 (81.8)	–	2 (18.2)	11 (100)
VII^b^	Category 3: Foods labeled as whole grain plus related terms	–	9 (81.8)	2 (18.2)	11 (100)

a Group I: Bakery goods, bread, cereals, and related products.

b Group VII: Sugars, sugary foods, and snacks.

In group VII, of the 11 foods in category 1, 9 (81.8%) exhibited whole-grain claims and 2 (18.2%) used the term as a claim and product name. Of the 11 foods belonging to category 3, 9 (81.8%) used whole grain as a product name and 2 (18.2%) as both claim and product name (Table 3).

Table 4 shows the analogous expressions displayed on the front-of-pack labels of processed and ultra-processed foods. The most common terms in group I were “whole-grain cereal” (31.0%) and “made with whole grains” (24.1%). In group VII, the most frequent analogous terms were “4 whole-grain cereals” and “7 whole grains” (6.9% each).

**Table 4:** Prevalence and examples of terms analogous to whole grain displayed on the front label of processed and ultra-processed foods based on cereals or pseudocereals, stratified into food groups according to Brazilian Resolution No. 359/2003.

**Analogous term**	**Prevalence in group I foods^a^**	**Examples of group I foods containing analogous terms**	**Prevalence in group VII foods^b^**	**Examples of group VII foods containing analogous terms**
	***n* (%)**		***n* (%)**	
Whole grain	18 (31.0)	Granola and breakfast cereal	–	–
Made with whole-grain cereals	14 (24.1)	Cereal bars, salted biscuits, and breakfast cereal	1 (1.7)	Sweet biscuits
100% whole-grain flour	4 (6.9)	Cake without filling and sliced bread	–	–
Produced with whole grains	3 (5.2)	Bread	–	–
With whole-grain flour	2 (3.4)	Cake without filling and bread mix	1 (1.7)	Cake with filling
Flour 100% whole-grain	2 (3.4)	Sliced bread	–	–
Five whole grains	2 (3.4)	Granola	–	–
With whole grains	1 (1.7)	Breakfast cereal	–	–
Produced entirely with whole-grain flour	1 (1.7)	Bread	–	–
With whole-grain rice	–	–	1 (1.7)	Corn snacks
Four whole-grain cereals	–	–	4 (6.9)	Cookies
Seven whole grains	–	–	4 (6.9)	Cookies
*N*	47 (81.0)	–	11 (19.0)	–
Total	58 (100)	

a Group I: Bakery goods, bread, cereals, and related products.

b Group VII: Sugars, sugary foods, and snacks.

The use of the term whole grain (category 1) on front-of-pack labels was significantly higher in group I than in group VII (Table 5).

**Table 5:** Prevalence of the term whole grain combined or not with analogous expressions on the front-of-pack label of processed and ultra-processed foods based on cereals or pseudocereals, stratified by food groups according to Brazilian Resolution No. 359/2003.

	**Category 1: Foods labeled** **as whole grain**			**Category 3: Foods labeled** **as whole grain plus related** **terms**		
**Food group**	**Without analogous expressions**	**With analogous expressions**	***p***	**Without analogous expressions**	**With analogous expressions**	***p***
	***n* (%)**	***n* (%)**		***n* (%)**	***n* (%)**	
I^a^	491 (85.0)	87 (15.0)		557 (96.2)	22 (3.8)	
VII^b^	414 (97.4)	11 (2.6)	<0.001^c^	418 (97.4)	11 (2.6)	0.276
Total	905 (90.2)	98 (9.8)		975 (96.7)	33 (3.3)	

a Group I: Bakery goods, bread, cereals, and related products.

b Group VII: Sugars, sugary foods, and snacks.

c Significant difference (Pearson's chi-squared test, p < 0.05).

**Figure 1:**
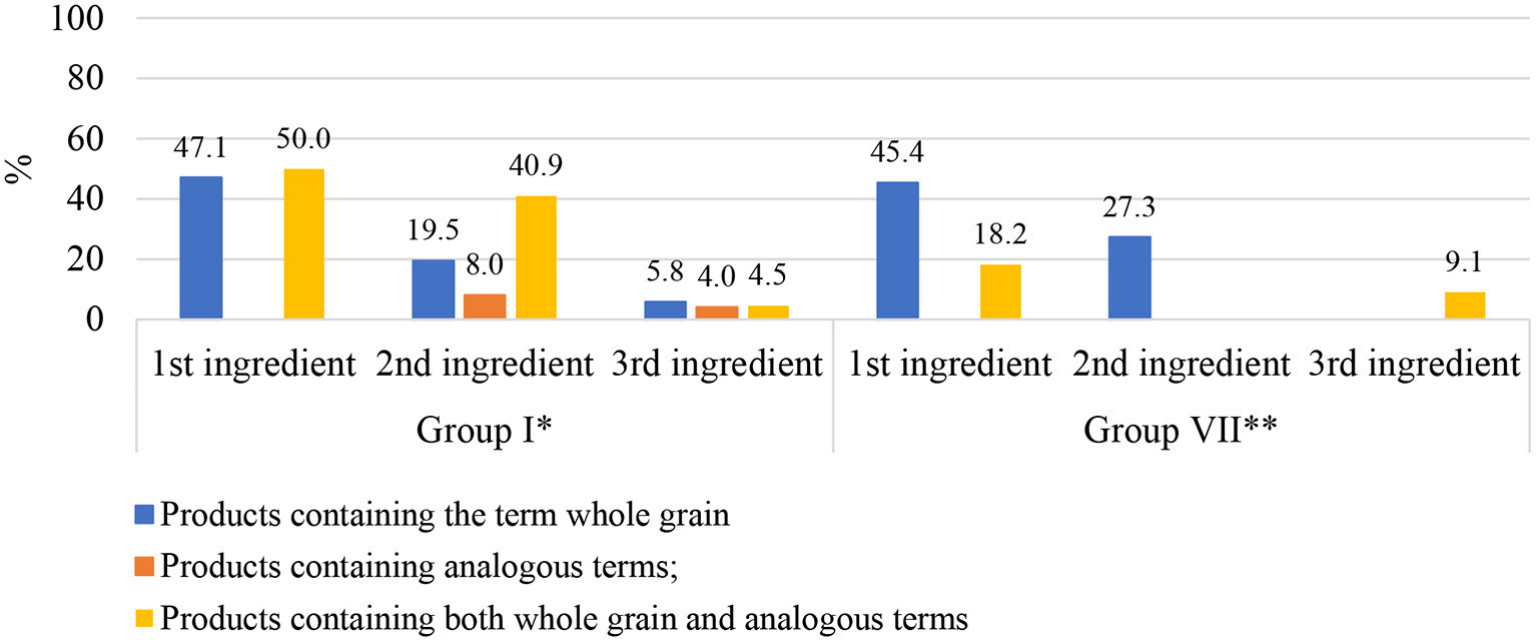
Position of whole-grain ingredients among the first three ingredients displayed in the ingredients list of processed and ultra-processed foods based on cereals or pseudocereals labeled as whole grain and/or related terms, stratified by food groups according to Brazilian Resolution No. 359/2003. *Group I: Bakery goods, bread, cereals, and related products. **Group VII: Sugars, sugary foods, and snacks.

Analysis of the ingredients list (Figure 1) showed that, in category 1 foods of group I, 47.1% (*n* = 41) of products had one type of whole grain as the major ingredient. For example, whole-wheat flour was the first ingredient of whole-wheat sesame biscuit. The frequency of products with whole grains as second (19.5%, *n* = 17) and third (5.8%, *n* = 5) ingredients was lower. None of the foods in category 2 had whole grains as the first ingredient, and only 12% (*n* = 3) had whole grains as the second or third ingredient. In category 3 foods, whole grains were the first ingredient in 50% (*n* = 11) of products, the second ingredient in 40.9% (*n* = 9), and the third ingredient in 4.5% (*n* = 1).

In group I, of the products that did not contain the term whole grain and/or analogous expressions (*n* = 444) on the front panel, 17 had a whole-grain cereal among the first three ingredients. In group VII, no product without the term whole grain and/or analogous expressions had a whole-grain cereal as one of the first three ingredients.

In group VII, 45.5% (*n* = 5) of products in category 1 had whole grains as the primary ingredient and 27.3% (*n* = 3) had whole grains as the second ingredient. In category 3, whole grain was the main ingredient in 18.2% (*n* = 2) of products and the second ingredient in 9.1% (*n* = 1).

## Discussion

The findings showed that 15.5% of processed and ultra-processed foods based on cereals and pseudocereals displayed the term whole grain or analogous expressions on front-of-pack labels. Furthermore, we observed a lack of standardization in the use of terms analogous to whole grains on front-of-pack labels. Studies analyzing food labels in Australia, Canada, and the United States of America (USA) found that 29, 21, and 20.7%, respectively, of breads, cereal products, and bakery products made reference to whole grains, displayed whole grain labels, or met the requirements to make whole-grain claims on the front-of-pack (36, 39, 57). The similarity between our results and those of the referred studies might be due to the lack of regulation on whole-grain foods in Australia, Brazil, and Canada (10, 39, 57, 58), which allows the food industry to use this type of information as a marketing tool. Studies have shown that, because consumers have little knowledge of whole grains, they might find it difficult to identify products containing these ingredients, thereby relying on food labels. In view of this, food labels that present clear information are useful to consumers at the time of purchase (39, 57, 58). It is worth noting that the presence of whole-grain ingredients does not necessarily imply good nutritional quality. Other nutrition information should be considered when assessing the healthiness of a product. For instance, in the study of Curtain and Grafenauer (59), it was found that grain-based muesli bars (*n* = 165) containing whole grains had higher energy, total fat, and dietary fiber values as well as lower sugar and sodium contents than bars with refined cereals. However, none of the muesli bars were considered low in sugar. Furthermore, in evaluating bars according to the classification of the Australian front-of-pack labeling system, whose objective is to help consumers make healthier choices within food categories, no significant difference was found between whole-grain and refined cereal bars.

In the current study, a higher prevalence of the term whole grain was found in the front panel of foods of group I than in the front panel of foods of group VII. This result was expected, given that foods of group VII had a lower amount of whole grains, as inferred by analysis of the ingredients list. The main characteristic of Sugars, sugary foods, and snacks (VII) is that they are ultra-processed, which, according to the literature, indicates a higher amount of refined flour.

According to the results obtained here, half of the food products labeled as whole grain did not contain a whole-grain cereal as the primary ingredient. Mozaffarian et al. (36), in analyzing the labels of cereal products sold in the USA, such as breads, cake, cereal bars, and cookies (*n* = 545), found that 40% of foods had whole grains as the first ingredient and 54.3% contained whole grains (regardless of the position in the ingredients list). In a Canadian survey assessing 436 labels of packaged breads, it was found that 65% of products contained at least one whole-grain ingredient and 24% contained one whole-grain ingredient in the first position of the ingredients list (39). Three studies conducted in Brazil assessing the labels of whole-wheat bread and biscuits (*n* = 21, *n* = 30, and *n* = 24) found that, among whole-wheat breads, 33.3 and 71.4% had whole wheat flour as the first ingredient, and 33.3% of whole-wheat biscuits declared whole-wheat flour as the first ingredient (37, 38, 40). Overall, the results of these studies are similar. Analysis of the ingredients list showed that products that displayed whole grain or similar terms on front-of-packs do not contain whole grains as the major ingredient, implying a lower amount of nutrients and phytochemicals. Thus, consumers might be being misled as to product composition.

A previous study in the USA aimed to determine criteria for identifying whole-grain products. The authors identified that information available on food labels could contribute to this end, including whole-grain claims and the presence of whole grains in the ingredients list, whether in the first or other positions. However, it was deemed that this information can confuse consumers and organizations, such as schools and cafeterias (36). The World Health Organization underscored, through the Global Strategy on Diet, Physical Activity, and Health, that consumers have the right to standardized, clear information on food composition for informed food choices (35).

Currently, for consumers to know whether a food product contains whole grains, it is necessary to consult the ingredients list, which is presented in descending order of weight (53). In Brazil, in view of the gap in whole-grain labeling legislation, the General Food Management (GGALI), together with ANVISA, conducted a study on the regulatory process of whole-grain foods (10). This investigation resulted in the publication of Resolution No. 493, April 2021, which came into force on April 22, 2022. The resolution determines the requirements of composition and labeling for cereal-based foods to be classified as whole grain and use whole-grain claims. According to the resolution, a food product is considered whole grain when at least 30% of ingredients are derived from whole cereals or pseudocereals and their amount is greater than that of refined ingredients. Foods may display the expression whole grain in their product name, provided that the total percentage of whole-grain ingredients be declared (47, 60). It should be noted that, in order to comply with the new legislation, food manufacturers must make changes to the way that information is presented on food labels, such as by disclosing the percentage of whole grains.

The limitations that arise from gathering data from one supermarket only must be considered. As the study was conducted in a large supermarket in southern Brazil, data may not reflect the profile of products sold throughout the country. Nevertheless, care was taken during supermarket selection to ensure that our database consisted of products of well-known brands that are found in other parts of the country.

To the best of our knowledge, this was the first study to analyze the use of whole grain and similar terms on the labels of processed and ultra-processed foods based on cereals and pseudocereals using a census database of food labels. The study is original and relevant within this field of research and may contribute to reducing the lack of publications in the area. Furthermore, our findings underscore the need to improve legislation and inspection, as well as to promote actions aimed at food and nutrition education to enable the population to understand and use food label information, thereby promoting informed food choices.

The results support the new changes in Brazil's label policy, such as the regulation proposed in 2021, which describes the composition requirements for labeling cereal-based foods as whole grain. These changes will help consumers to easily and quickly identify a whole-grain product during food purchase, allowing comparisons between products within the same food category. Such information may positively stimulate Brazilian consumers to minimize the consumption of products that do not meet their expectations or necessities with regard to whole-grain foods.

## Conclusion

Our results showed that, of the 1,004 processed and ultra-processed food products based on cereals and pseudocereals analyzed, 9.8% had the term whole grain on the front-of-pack, 2.5% exhibited analogous terms, and 3.3% used both whole grain and analogous terms. This finding highlights the need to regulate whole-grain labeling to ensure clear and standardized information so as not to confuse consumers.

In assessing the position of whole-grain ingredients on the ingredients list, we found that about half of all food products analyzed did not have a whole-grain cereal as the primary ingredient. It can be said that cereal- and pseudocereal-based foods that display whole grain or similar terms on the front-of-pack contain inaccurate, unclear, or misleading information, which may prevent consumers from making informed food choices.

The results of this study may contribute to the strengthening of public policies aiming to improve food labeling legislation in Brazil. This research is relevant to show that, before implementation of regulatory requirements for whole-grain labeling, at least half of the foods containing cereals were inappropriately identified as whole grain. Thus, our results underscore the importance of this new regulation to improve the quality of information on whole foods available to consumers and its potential to improve the nutritional quality of cereal- and pseudocereal-based foods in Brazil. Future studies can be conducted to assess changes in the nutritional quality of cereal- and pseudocereal-based foods with different degrees of processing and investigate whether marketing strategies promote the consumption of healthier foods.

## Data availability statement

The raw data supporting the conclusions of this article will be made available by the authors, without undue reservation.

## Author contributions

ÉB was responsible for planning the research, collecting, analyzing, interpreting the data, and drafting the manuscript. PU, AN, and AG were responsible for planning the research, collecting, analyzing, and interpreting the data, and revising the manuscript. AF and GB contributed to data interpretation and manuscript revision. RP was responsible for designing the original study, planning research and analysis, coordinating the research, supervising, revising the final manuscript. All authors approved the version submitted for publication.

## Funding

This study was conducted as part of a wider study on the comprehension and use of food labels in Brazil, funded by the National Council for Scientific and Technological Development (CNPq) of the Brazilian Ministry of Science and Technology and by the Brazilian Health Regulatory Agency (ANVISA) (grant number 440040/2014-0), with the aim of filling gaps related to policies, management, and organization of the Brazilian National Health System. The funders had no role in the design, analysis, or writing of this article.

## Conflict of interest

The authors declare that the research was conducted in the absence of any commercial or financial relationships that could be construed as a potential conflict of interest.

## Publisher's note

All claims expressed in this article are solely those of the authors and do not necessarily represent those of their affiliated organizations, or those of the publisher, the editors and the reviewers. Any product that may be evaluated in this article, or claim that may be made by its manufacturer, is not guaranteed or endorsed by the publisher.
