# Supplementary figures and images for: Use of the term whole grain on the label of processed and ultra-processed foods based on cereals and pseudocereals in Brazil
Source: Front Nutr. 2022 Aug 15;9:875913. doi: 10.3389/fnut.2022.875913 (PMC9421291; doi:10.3389/fnut.2022.875913)

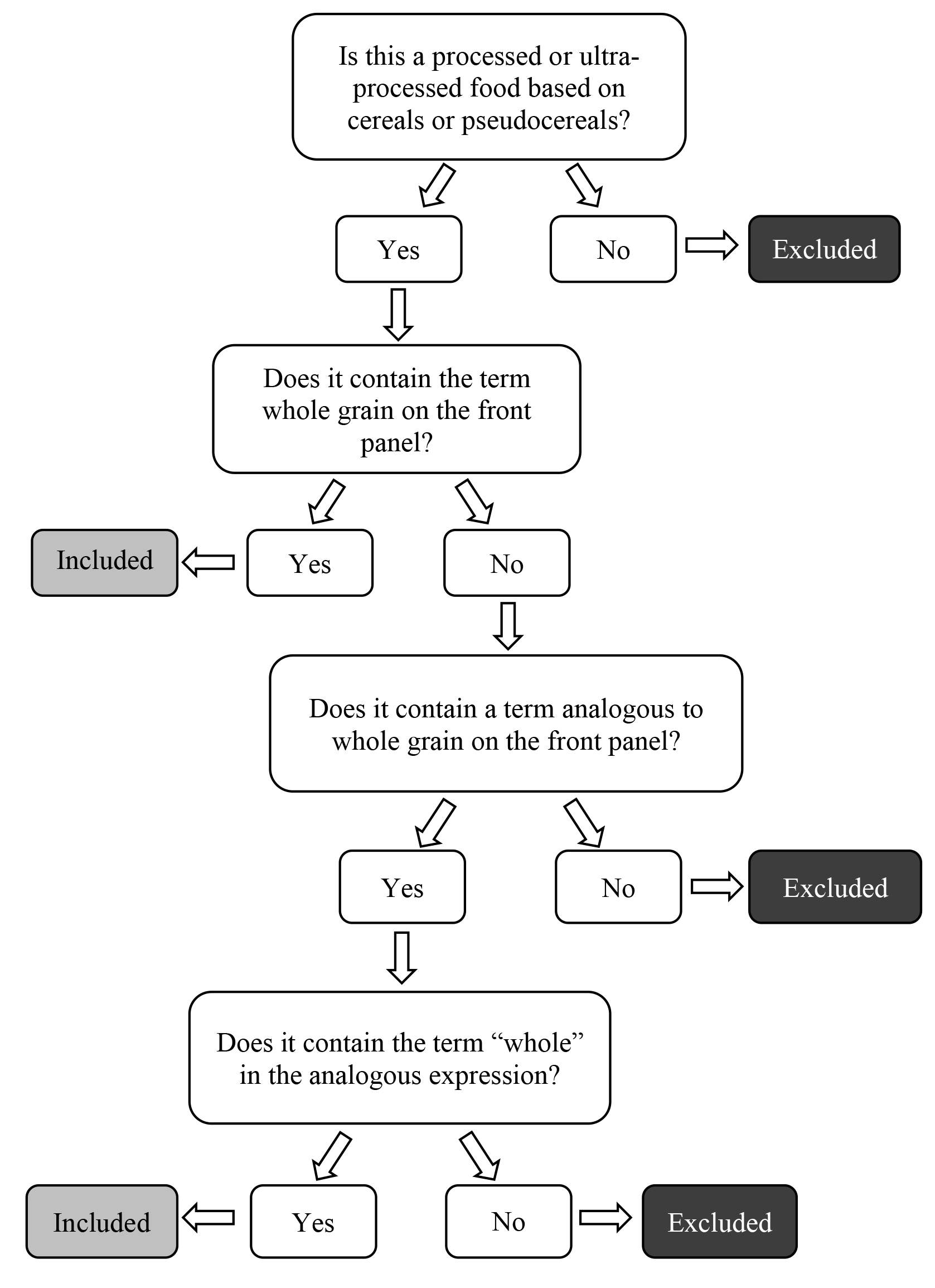

Supplement: Supplementary file 1 [file Image_1.TIF]
